# Supplementary material for: AI Aided Design of Epitope-Based Vaccine for the Induction of Cellular Immune Responses Against SARS-CoV-2
Source: Front Genet. 2021 Mar 25;12:602196. doi: 10.3389/fgene.2021.602196 (PMC8027494; doi:10.3389/fgene.2021.602196)
Supplement: Supplementary file 3 [file Presentation_1.pdf]

Supplementary Materials 1

Mutation frequency for each position within all SARS-CoV-2 proteins.

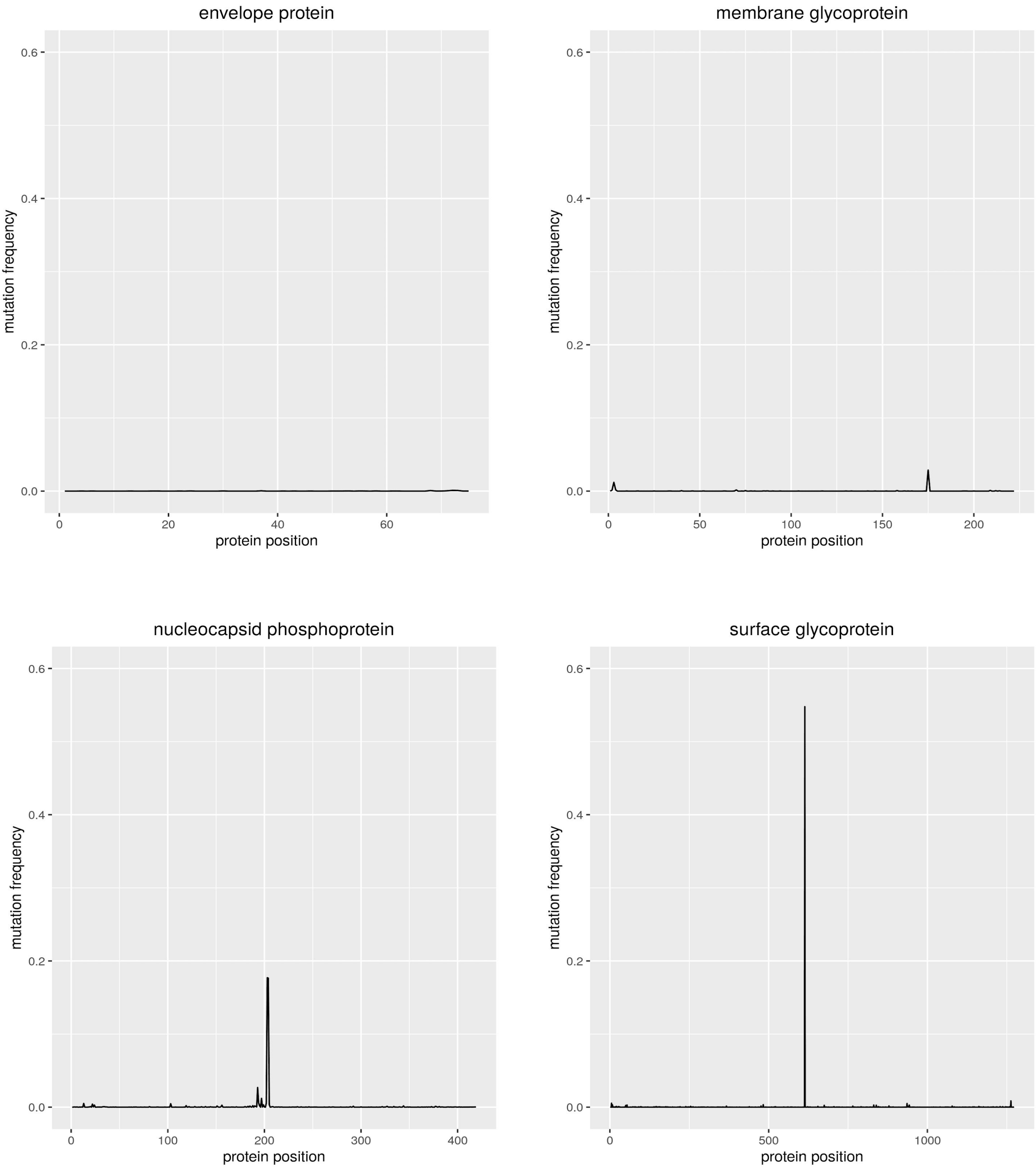

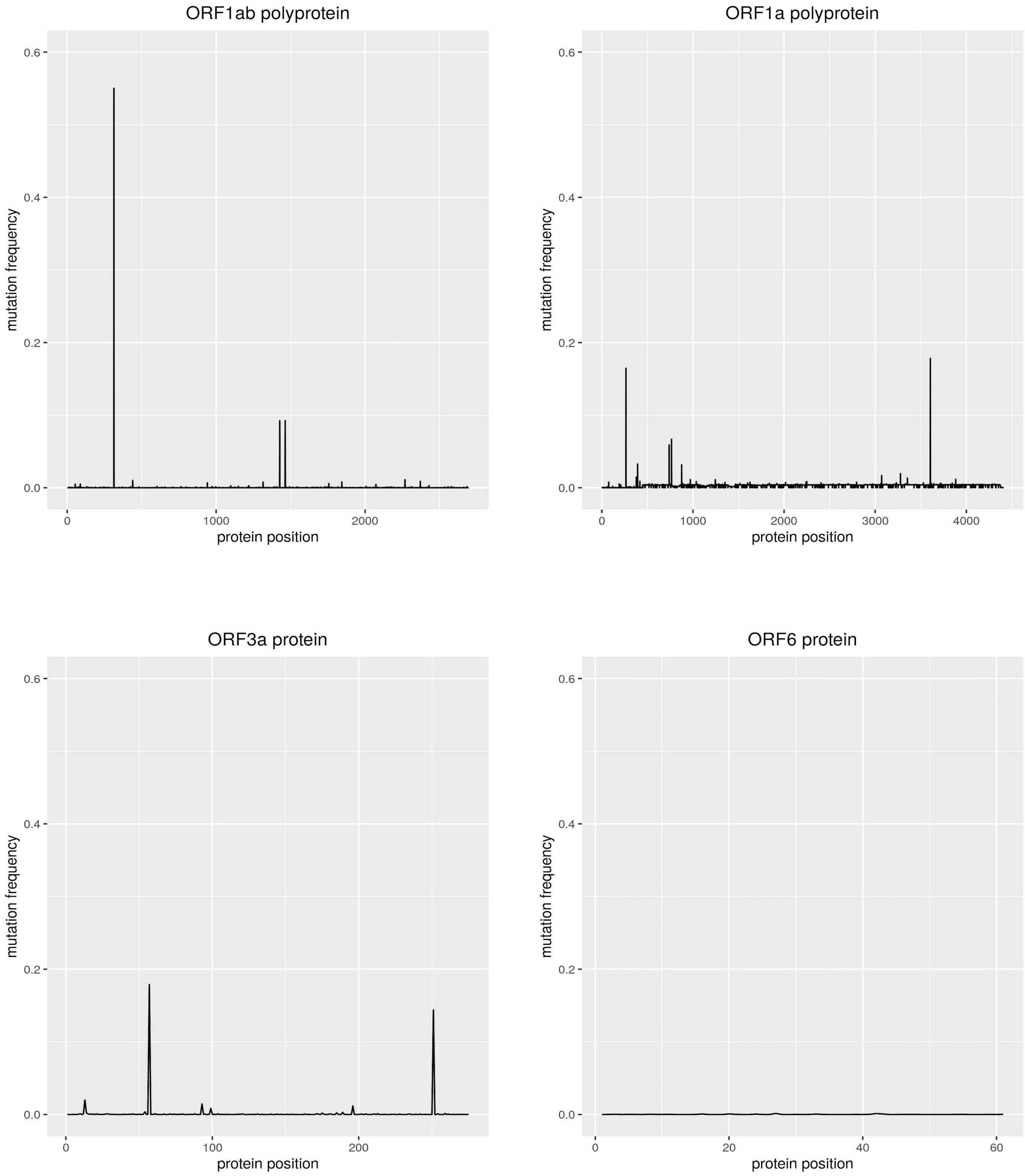

ORF1ab polyprotein
mutation frequency
protein position
0.0
0.2
0.4
0.6
0
1000
2000
ORF1a polyprotein
mutation frequency
protein position
0.0
0.2
0.4
0.6
0
1000
2000
3000
4000
ORF3a protein
mutation frequency
protein position
0.0
0.2
0.4
0.6
0
100
200
ORF6 protein
mutation frequency
protein position
0.0
0.2
0.4
0.6
0
20
40
60

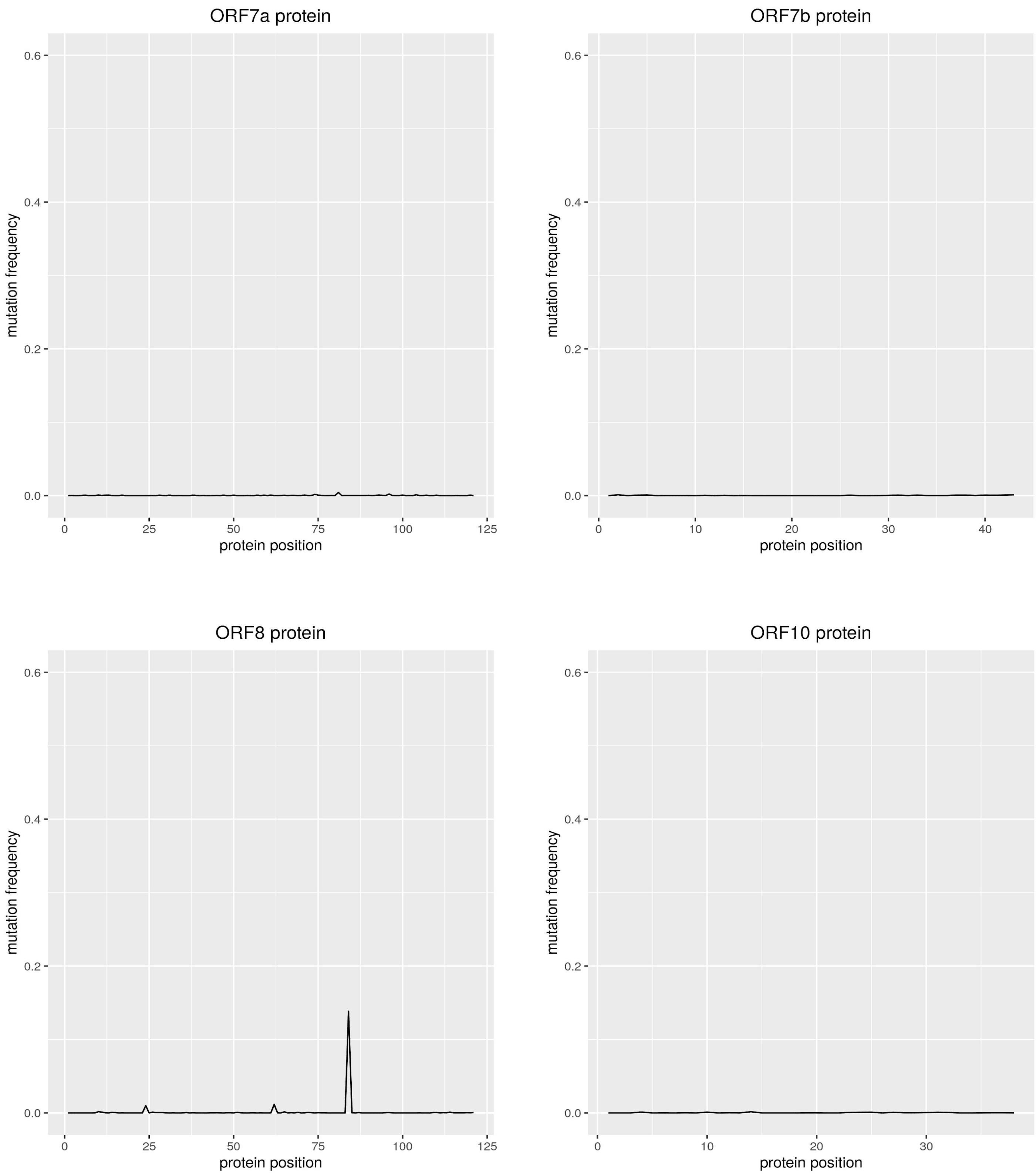

ORF7a protein
ORF7b protein
ORF8 protein
ORF10 protein
mutation frequency
protein position
0.0
0.2
0.4
0.6
0
25
50
75
100
125
10
20
30
40
